# Messaging and Information in Mental Health Communication on Social Media: Computational and Quantitative Analysis

**DOI:** 10.2196/48230

**Published:** 2025-07-03

**Authors:** Rebecca K Ivic, Amy Ritchart, Shaheen Kanthawala, Heather J Carmack

**Affiliations:** 1 College of Communication and Information Sciences, University of Alabama, 901 University Ln, Tuscaloosa, AL, 35401, United States; 2 Department of Journalism & Creative Media, University of Alabama, Tuscaloosa, AL, United States; 3 Kern Center for the Science of Health Care Delivery, Mayo Clinic, Rochester, MN, United States

**Keywords:** health communication, mental health, mental health organizations, computational analysis, LIWC, Linguistic Inquiry and Word Count, large scale data

## Abstract

**Background:**

Mental health organizations have the vital and difficult task of shaping public discourse and providing important information. Social media platforms such as X (formerly known as Twitter) serve as such communication channels, and analyzing organizational health information offers valuable insights into their guidance and linguistic patterns, which can enhance communication strategies for health campaigns and interventions. The findings inform strategies to enhance public engagement, trust, and the effectiveness of mental health messaging.

**Objective:**

This study examines the predominant themes and linguistic characteristics of messages from mental health organizations, focusing on how these messages’ structure information, engage audiences, and contribute to public information and discourse on mental health.

**Methods:**

A computational content analysis was conducted to identify thematic clusters within messages from 17 unique mental health organizations, totaling 326,967 tweets and approximately 7.2 million words. In addition, Linguistic Inquiry and Word Count (LIWC) was used to analyze affective, social, and cognitive processes in messages with positive versus negative sentiment. Differences in sentiment were assessed using a Mann-Whitney *U* test.

**Results:**

The analysis revealed that organizations predominantly emphasize themes related to community, well-being, and workplace mental health. Sentiment analysis indicated significant differences in affect (*P*<.001), social processes (*P*<.001), and cognitive processing (*P*<.001) between positive and negative messages, with effect sizes that were small to medium. Notably, while messages frequently conveyed positive sentiment and social engagement, there was a lower emphasis on cognitive processing, suggesting that more complex discussions about mental health challenges may be underrepresented.

**Conclusions:**

Organizations use social media to promote engagement and support, often through positively valanced messages. Yet the limited emphasis on cognitive processing may indicate a gap in how organizations address more nuanced or complex mental health issues. Findings demonstrate the need for communication strategies that balance information with depth and clarity, ensuring that messages are trustworthy, actionable, and responsive to multiple mental health needs. By refining digital messaging strategies, organizations can enhance the effectiveness of health communication and improve engagement with mental health resources.

## Introduction

### Background

Mental health conditions represent an urgent and escalating global public health crisis. The burden of mental illness is global, yet structural barriers, including underfunded resources and lack of access to care, persist across regions and economic contexts. The World Health Organization’s (WHO) Comprehensive Mental Health Action Plan 2013‐2030 emphasizes the need for coordinated, scalable interventions, but a widening treatment gap remains, particularly in low-resource settings [1]. In this landscape, digital platforms serve as important places for mental health information, offering opportunities for intervention, advocacy, and public engagement at a scale typically unattainable through traditional health communication channels. Organizational structures play a vital role in shaping public discourse and mobilizing resources through social media [2-5]. They have the potential to facilitate large-scale mental health campaigns using health communication strategies. As the global mental health crisis intensifies, it is critical to understand how organizations use digital platforms inform and build trust and ensure that mental health messaging is clear, accessible, and actionable.

### Mental Health Organizations on Social Media

Health communication campaigns through advocacy strategies have been identified as a possibility for positive change for people experiencing these challenges [6]. The WHO called for key organizations to assist with mental health issue resolutions [7]. Similarly, the Center for Disease Control and Prevention put forth guidelines on health communication through text-based social media platforms like Facebook (Meta Platforms) or X (formerly known as Twitter) [8]. The importance of messages from such organizations has been examined in past research. Smith-Frigerio [6] examined 2 mental health advocacy groups’ social media strategy. She found their messaging included awareness-raising strategies, support of various policy initiatives, and the promotion of diversity and inclusivity. Another study followed 3 mental health organizations’ messages and engagement for a year and noted that content focused on mental health received more engagement as compared to relationship building or event promotion [9]. When investigating visual social media posts, Jia et al [10] noted that posts depicting knowledge about mental health disorders, their treatments, and antistigma frames were more heavily engaged content.

To better understand how mental health organizations communicate effectively, this study applies the Crisis and Emergency Risk Communication (CERC) framework to analyze the affective, social, and cognitive dimensions of their messaging. CERC helps organizations craft communication that enhances sensemaking and efficacy, ensuring messages are clear, actionable, and trustworthy. Traditionally used in public health crises [11,12], CERC is relevant to mental health communication, where strategic messaging can reduce stigma, build trust, and improve access to care [1,7]. This study extends CERC’s application to social media, analyzing how mental health organizations engage with these principles in digital discourse.

### Health Communication and Computational Text Analysis

A body of research has investigated the use of computational qualitative analysis in a broad range of disciplines, including health communication [12], psychology [13], tourism [14,15], physician education [5,16], and other domains. Importantly, we recognize there are advantages and disadvantages when using such approaches. Leximancer, for instance, was used [17] to strengthen the validity of their study of Indigenous voices in a public health curriculum. Gibson et al [18] examined a large volume of social media data regarding e-cigarette and tobacco coverage, using automated coding to content analyze the data. To generate reliability in the content analysis, they calculated the consistency of reliability for weekly estimates with a threshold set to >.70. Based on this, they found that X and YouTube (Alphabet Inc) contained fewer themes compared to other data sources. Vos and Buckner [11] examined an emerging crisis about the H7N9 virus and messages, drawing upon the CERC framework to address the ongoing communication that happened at various stages of the crisis. To analyze 25,598 unique messages, automated coding was used to identify sensemaking and efficacy messages in messages.

Social media sites can have a profound effect on communication regarding mental health, but there remains a dearth of knowledge on the detection of concepts and themes in a larger corpus of mental health discourse, especially from the perspective of official organizations, though this technique has been used in other fields, such as digital entrepreneurship [19,20]. Likewise, the application of Leximancer to health issues research is recent. There is a compelling opportunity for scholars to use automated text analysis to examine health-related data, especially when other approaches cannot process the volume of data. Considering this, automated data analysis to quickly understand the corpus of large amounts of words and text in data can be useful in health research, although it is not a panacea for understanding phenomena [21].

In prior research, these approaches have been used to explore larger data sets and mental health but have yet to fully emerge in health communication-related research, though they have been explained using interpretivist approaches from survey data with larger response rates (n=934; [22]). Other studies [23] investigated the environmental effects of COVID-19 using Leximancer. They first used a segmented regression technique and then applied automated content analysis on environment-related subreddits to gain insight into the evolution of risk amplification and ripple effects. In this study, Leximancer was used to demonstrate how large data sets may be used in qualitative research in the context of health communication, specifically in nuanced communication through mental health. The capabilities regarding automated textual analysis and interactive, visual depictions of the relationships among the data have long been highlighted as important for extracting the meaning of themes. Examining the predominant themes in mental health communication can yield important insights into the issues and challenges organizations face in disseminating and ultimately reaching the public.

The following research question is offered:

Research question 1 (RQ1): What are the most prevalent themes present in messages about mental health led by mental health organizations?

### Message Sentiment

Linguistic features are integral to shaping the themes expressed in communication, which is an inherently complex process. As a fundamental component of communication, language influences how messages are interpreted and significantly impacts psychological health [24]. Linguistic Inquiry and Word Count (LIWC) has long been used in research to understand cognitive complexities in language use and texts, including social media data, and in relation to mental health. Research has indicated that depressed patients have been more likely to use negative emotion words and express more self-focused thoughts [25], while the use of types of words, such as causation words and self-discrepancies, have been linked to positive health outcomes [26]. Therefore, to analyze language use in the messages from mental health organizations in terms of their affect, social, and cognitive processes using LIWC, this research adds deeper value into the study of the relationship between mental health and communication. A total of 3 fundamental processes are analyzed: affect (emotional contents of language), social (process of interactions with others), and cognitive processes (how information is processed and language regarding insights, causation, and discrepancies).

Social media platforms serve as key sources of mental health information, offering vital insights into how organizations communicate and engage the public. Researchers have increasingly used LIWC to analyze mental health–related content on X, examining linguistic patterns and their role in shaping information delivery. This study explores how mental health organizations structure their messaging within a large text corpus. Based on this, we propose the following hypothesis (H1):messages from mental health organizations will vary such that those with positive sentiments will have significant differences in affect, social, and cognitive processes compared to those with negative sentiments.

## Methods

### Data Collection

Tweets from mental health care organizations were collected using the Twitter API using March 2023 as a deliberate sampling frame due to the data size and scope. The organizations were identified through a snowball sampling approach, which began with accounts that were reshared or followed by a population of relevant accounts. The goal was to compile a list of accounts explicitly focused on mental health promotion or services or for which mental health was a core organizational value. Queries were conducted using organization names and their common abbreviations with specific parameters (eg, NIMHgov for the National Institutes of Mental Health), following methods of previous studies [27]. The initial sample included accounts reshared or followed by relevant organizations, and the iterative nature of the snowball sampling allowed the sample to expand gradually. This process resulted in a final dataset of 17 unique accounts queried. The dataset includes 326,967 tweets from 17 unique mental health organizations (see Multimedia Appendix 1). This dataset consists solely of tweets originally posted by these organizations and does not include mentions, replies, or retweets from other users. Account-level metadata, such as follower count and creation date, was not collected as part of the extraction process and is not available for analysis. Sampling procedures and account inclusion criteria were consistent with established scholarship [27]. Multimedia Appendix 1 provides a list of the accounts, including their X handles, full names, and the number of messages they posted at the time of data collection.

Data were extracted from JSON files and saved as a CSV file to facilitate analysis and ensure alignment with the study’s focus on mental health communication. The dataset included details such as the text of each post, post type (eg, social media engagement metrics such as likes, quote counts, replies, and retweets), timestamp, and number of impressions. To assess the extent of irrelevant content, a review was conducted on a randomly selected 10% (32,900) subset of tweets. Following this review, thematic extraction was refined using Leximancer’s automated procedures. To minimize data noise, the thematic relevance threshold was adjusted to retain only central, frequently co-occurring concepts in the final analysis. Concepts with weak connectivity, such as isolated fundraising messages, promotional event announcements, or generic engagement phrases (“join us” or “support this”), were ranked lower and excluded from core thematic groupings. Leximancer identified thematic clusters through semantic co-occurrence analysis, assigning prominence scores using a color-coded red, green, and blue (RGB) scale. Concepts most frequently appearing in central discourse were highlighted in hotter colors (red, orange, and yellow), while less prominent or potentially irrelevant content was represented in cooler colors (green, blue, and violet).

### Ethical Considerations

We were mindful in our use of publicly available social media data, particularly given the ethical complexities surrounding mental health content. Ethical standards in this area are still evolving, and while many users may have awareness that their data can be accessed under terms and conditions, the extent of that understanding likely varies considerably [28,29]. We removed any potentially identifying information from the dataset, and usernames and IDs were not included in the queried API. As a result, the dataset only consisted of the aforementioned information, and no personally identifying information was included in the data analysis. We recognized that some groups may be either under- or overrepresented when analyzing the themes and sentiment in a larger scale analysis, and in addressing a stigmatized health issue [30]. We were cognizant of the potential harms and risks and sought to be mindful of these in the analysis by analyzing only text contents and excluding any user IDs or potentially identifying information.

### Data Analysis

RQ1 was analyzed using Leximancer, an automated machine learning software that identifies thematic clusters without requiring predefined coding categories. Leximancer detects co-occurring terms within a dataset, using semantic relationships rather than word frequency alone to reveal conceptually connected themes [31]. This approach enables researchers to analyze patterns in large text corpora by grouping related ideas based on their contextual associations. While Leximancer generates data visualizations that organize key concepts into thematic clusters, researcher interpretation remains essential [3,13]. In these visualizations, themes are named based on the most prominent concept within each cluster, and researchers can refine these labels by reviewing associated terms. The strength of each concept is visually represented through a color gradient, ranging from red (most prominent) to violet (least prominent), reflecting its frequency and relevance within the data set.

Concept maps were generated using Leximancer to identify key topics emerging from the messages. Standard semantic and relational extraction techniques were applied. In the semantic extraction phase, the software identified fundamental concepts, terms that frequently appear together, by analyzing their frequency, occurrence, and co-occurrence. These concepts were then weighted and placed in a co-occurrence matrix, with a thesaurus of highly relevant words and phrases generated to refine semantic meaning [32]. In the relational extraction phase, the discovered concepts were analyzed to determine their associations. After computing concept count, co-occurrence frequency, and relative importance, a data visualization was created. This visualization prioritizes highly co-occurring concepts, clustering them into themes based on their strongest connections.

To address H1, LIWC was used to measure the emotional tone of messages made, comprising roughly 7.2 million words and the full 326,967 tweets. We computed the mean score, SD, skewness, and kurtosis for each dimension of affect (mean 0.111, SD 0.079; skewness=1.516, kurtosis=5.171), social (mean 0.1752, SD 0.1055; skewness=0.973, kurtosis=1.838), and cognitive (mean 0.094, SD 0.077; skewness=0.914, kurtosis=1.374). Out of each of the dimensions, the distribution of affect scores was found to be significantly left-skewed (*z*=−250.589; *P*<.001), indicating that most mental health organizational messages with negative sentiment held higher affect scores compared to those with positive sentiment. Likewise, because messages were made by distinct mental health organizations and presumably different users (thus treating cases as independent observations), a Mann-Whitney *U* test was conducted.

## Results

### Overview

**Figure 1.**
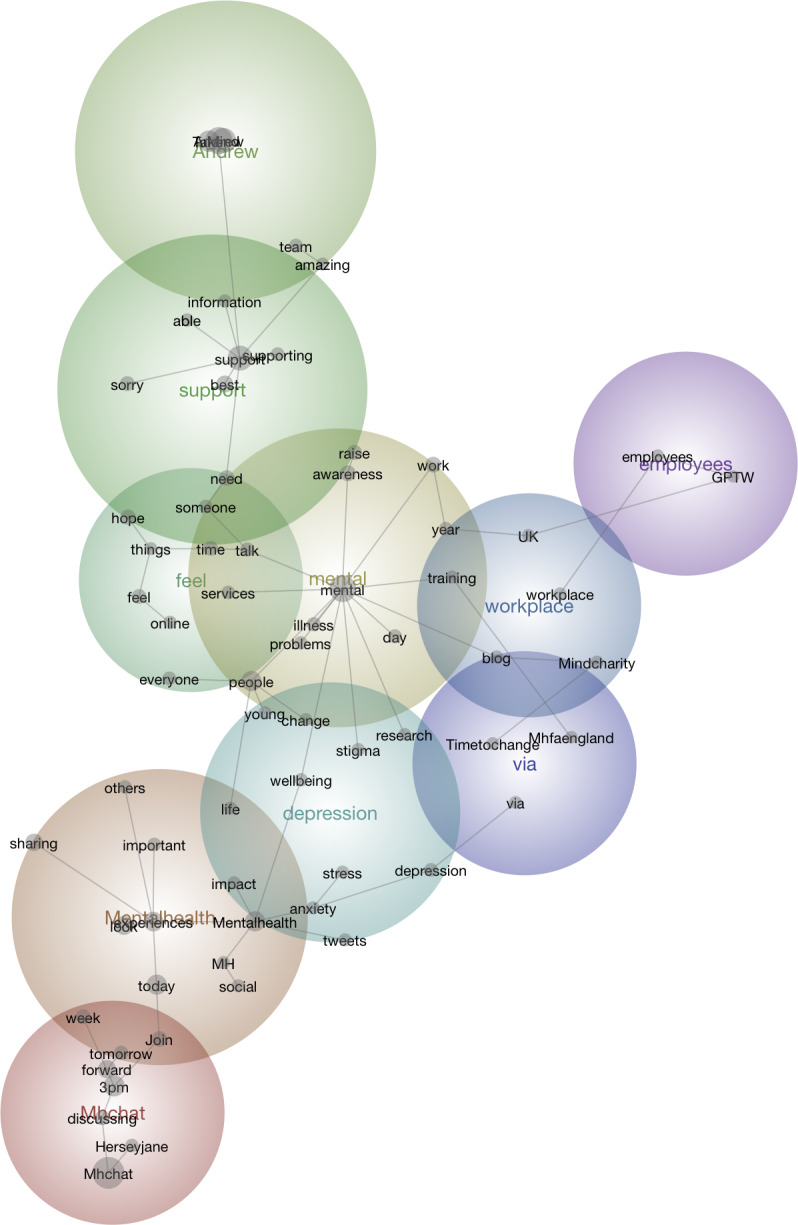
Concept map of the data.

The findings are presented by first addressing the results of RQ1, followed by H1. RQ1 focused on identifying the most prevalent themes in messages about mental health shared by mental health organizations. The concept map output, which considers the dataset regardless of the date or time of messages, revealed a y-shaped structure with 2 distinct paths, where the left path is slightly more prominent than the right. Figure 1 shows the concept map. Following recommendations for conducting research with Leximancer [32], we identified the dominant concepts within these clusters as themes, including example messages were relevant. Leximancer identified thematic clusters based on semantic co-occurrence patterns in the dataset. The most prominent themes, community, mental health, and well-being, emerged without predefined coding categories. These themes were later interpreted through the lens of CERC, which emphasizes trust-building, efficacy, and social support. While Leximancer does not apply theoretical labels, the resulting themes align with CERC principles, reinforcing its relevance to digital mental health messaging and are later included in the discussion.

**Figure 2.**
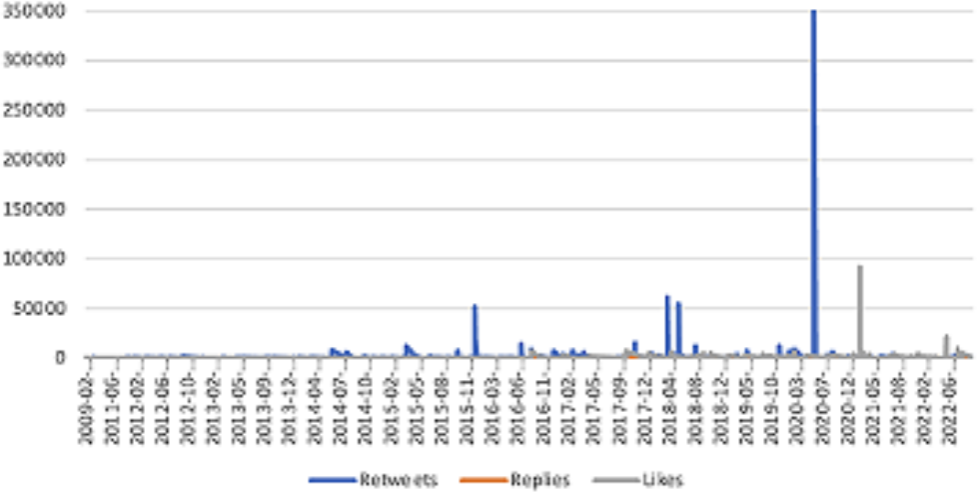
Temporal engagement over time with mental health messages.

A time series analysis further highlights the evolution of engagement with the message data (Figure 2). The y-axis represents the total number of engagements, including likes, retweets, and replies, while the x-axis denotes the date and time of posts. This analysis illustrates how engagement fluctuated over time, with periods of heightened activity aligning with significant mental health-related events. For example, the largest spike in activity occurred in March 2020, marked by a surge in retweets, coinciding with the WHO’s declaration of COVID-19 as a global pandemic.

### RQ1 Findings

The most prominent thematic cluster identified was community, followed by mental health and well-being. The first 2 clusters demonstrated a linear relationship, where topics associated with community were directly linked to mental health, without further connections to other themes in the concept map. In contrast, the discourse surrounding mental health extended to less prominent, peripheral themes such as depression. Although these themes were less centralized, they held notable importance within the overall communication landscape. For example, some messages highlighted calls to action, such as: “What are you doing for your mental health this holiday season?” Posts such as these were often accompanied by links to resources promoting self-care and mental health practices, encouraging individual engagement with mental health management.

While well-being was not the most dominant theme, it served as a crucial connecting point in the y-shaped conceptual structure, bridging otherwise distinct clusters. The theme of well-being acted as a connecting point, linking concepts such as feelings, social support, and Twitter users. This indicates that mental health organizations strategically promoted prosocial messaging aimed at fostering shared communication and engagement. Many messages sought to directly interact with audiences through participatory activities, such as fundraising initiatives or encouraging retweets. In some cases, these messages attempted to challenge public perceptions of mental illness, as illustrated by posts like: “What does mental illness look like to you? [...] Send yours to [fundraising, redacted].” In addition, organizations shared practical resources designed to assist individuals in need, including helplines and support services. For instance, messages advertised emotional support systems: “Mentalhealth helpline offering specialist emotional support, guidance, and information” and “Textcare provides emotional support and connection when you need it most.”

In the concept map’s right-side path, less prominent themes emerged, including workplace and employees. These themes illustrated the role of work environments in shaping mental health and well-being, with organizations sharing messages emphasizing the importance of supportive workplace cultures. For example:

Organisational culture is critical to #wellbeing in the #workplace” linked to reports outlining strategies for fostering mental health in professional settings. Similarly, some messages underscored the value of positive work environments with messages such as, “Creating a great work environment matters. #healthyworkplace.

Although themes on the periphery of the concept map, such as employee were ranked as less prominent, they provided meaningful insights into specific facets of the discussion. For example, the employee cluster included terms like “GPTW” (Great Place to Work), which may strengthen messaging about workplace mental health. These findings suggest that peripheral themes, while less frequent, often enrich the central discourse by adding nuanced perspectives and addressing less commonly explored areas.

These thematic clusters reflect a comprehensive strategy by mental health organizations to engage diverse audiences. Dominant themes serve as anchors, while secondary and peripheral themes act as bridges, connecting audiences to lesser-explored topics. These layered insights not only enhance the understanding of mental health communication but also highlight opportunities for expanding outreach and improving messaging strategies. The implications of these findings are explored further in the discussion.

### H1 Results

H1 examined whether there would be a significant difference in emotional processes in mental health organizational messages with positive sentiment compared to negative sentiment. LIWC was used to measure sentiment-related linguistic features, including affect, social, and cognitive processes. This analysis was conducted independently of Leximancer and was not used to categorize themes. A Mann Whitney-*U* test was performed, which showed significant differences in affect (*U*=6.582E+9, *z*=−250.589; *P*<.001), social processes (*U*=7.582E+9, *z*=−213.367; *P*<.001), and cognitive processes (*U*=1.275E+10, *z*=−21.660; *P*<.001) between mental health messages with positive sentiment compared to those with negative sentiment. The effect sizes for these differences were small (cognitive: *r*=−.205) to medium (affect: *r*=−.556, social: *r*=−.469).

These results suggest that the mean rank of messages containing negative tone were altogether lower than mean rankings of messages that were positive in tone. Thus, although the negative effect sizes suggest that each of affect, social, and cognitive dimensions held lower means, the values were highly statistically significant. Therefore, we suggest the results may support H1, which predicted differences in each of these processes in the emotional tone in messages.

## Discussion

### Principal Findings

Effective health communication goes beyond simply raising awareness; it requires transparency, credibility, and a commitment to delivering high-quality health information that resonates across different populations. This study underscores the essential role of health communication in shaping public perceptions of mental health and ensuring that information shared by organizations is accessible, trustworthy, and actionable. These findings highlight how mental health organizations use social media to frame information around well-being, but they also reveal gaps in addressing structural barriers to care and the need for more nuanced engagement strategies.

The analysis reveals that mental health organizations primarily emphasize community, well-being, and support networks in their messaging. While these themes foster positive engagement, there is a pressing need for clearer, more actionable communication strategies that go beyond broad encouragement to provide concrete guidance. Effective messaging must acknowledge the complexities of mental health struggles, offer practical pathways to care, and address the nuances of different lived experiences. Trust remains central to mental health communication, and organizations must ensure their messaging is both scientifically accurate and culturally responsive to reach audiences effectively.

Regarding H1, the linguistic analysis further underscores the role of emotion and social connection in digital mental health messaging. Findings indicate that while positive sentiment is a dominant strategy, it may not always align with the lived experiences of individuals facing mental health challenges. Overly optimistic framing, while engaging, could inadvertently dismiss the complexity of mental health struggles, or discourage open conversations about distress. A balance is needed: one that promotes hope while validating the realities of mental health needs. Health communication efforts must consider how organizations can refine their language to be impactful, ensuring that health information is framed in ways that build trust, encourage help-seeking, and reduce stigma.

These findings align with the CERC framework, which emphasizes that effective health communication should engage and provide clear, actionable information that enables individuals to respond to their circumstances [11,12]. While organizations successfully foster social connection, many messages lack the informational clarity and efficacy-building components that CERC underscores as essential in public health communication. Without concrete guidance, audiences may struggle to translate engagement into action. Applying informed strategies, such as reinforcing practical coping strategies, clear pathways to care, and guidance on recognizing and addressing mental health concerns, can help organizations move beyond awareness-building toward delivering information that empower individuals to take meaningful steps in managing their health.

Mental health communication takes place globally on social media platforms, with differing social and cultural contexts that shape how messages are received and interpreted [33]. As mental health organizations engage with global audiences, understanding how language functions across affective, social, and cognitive dimensions [34] are essential for researchers and practitioners. However, while fostering a positive emotional tone can enhance engagement, it is equally important to recognize that cultural variations in emotional expression and information shared may lead to unintended consequences. Overemphasizing positivity may inadvertently invalidate negative emotions, reinforcing stigma or limiting dialogue about health struggles, particularly in areas where stigmatization is an issue. To maximize impact, organizations must ensure that their messaging is culturally responsive, contextually appropriate, and globally inclusive of lived experiences.

By identifying key themes and emotional tones that effectively engage audiences, researchers can guide the development of evidence-based communication strategies for mental health organizations [10,35]. The study’s use of computational analysis alongside LIWC demonstrates a scalable approach to analyzing social media data, offering insights that extend beyond mental health to broader health communication. These findings provide organizations with actionable strategies to craft messages that are informative and impactful, ensuring they resonate with audiences encourage engagement.

These results highlight the important role of language in shaping mental health communication through information shared on social media, particularly the impact of positive sentiment on affective and social processes. The lower cognitive processing scores observed in mental health-related messages suggest that individuals facing mental health challenges may find it difficult to engage in complicated reasoning or articulate their experiences fully. This has important implications for organizations aiming to craft messages that are both accessible and solutions oriented. Rather than relying solely on positivity, messaging should balance encouragement with clarity, offering tangible strategies that individuals can apply to maintain or restore wellness. Findings from H1 reinforce the need for a deeper understanding of how language influences engagement with mental health communication on social media. A more nuanced approach to message design, grounded in both scientific accuracy and sensitivity to lived experiences, can enhance the effectiveness of mental health discourse, ensuring it supports those who need it most.

### Limitations and Future Directions

This study examined overarching thematic patterns in mental health communication across organizations, rather than stratifying by account type. While differences in messaging strategies may exist between government agencies, advocacy groups, and service providers, our analysis was designed to capture broad trends rather than organizational distinctions. Future research could explore these variations by applying stratified thematic analysis. This study builds on previous scholarship while recognizing its potential limitations [36].

A limitation of this study is that themes were analyzed at the aggregate level without stratifying by organization type. Future research could examine whether certain themes are more prominent among different categories of mental health organizations, providing additional insights into institutional messaging strategies. Using X as a data source means that the sample reflects conversations from a public stream, which may introduce biases based on the users who actively participate on the platform [36]. Automated sentiment analysis, while valuable, has inherent limitations in capturing the full nuance and complexity of human emotions. Although emotional tone can be quantitatively measured, it may not fully account for the range of interpretations and contextual meanings in messages. Furthermore, snowball sampling was used to identify relevant accounts by tracking those frequently retweeted or followed. This method ensured consistency in the dataset but may have led to overrepresentation of highly visible accounts, potentially overlooking lesser-known organizations and users. Social media research also faces broader challenges, as the nature of online information and messages varies across platforms. Differences in audience demographics, communication styles, and modes of expression highlight the importance of comparing discussions across multiple platforms to better understand the specific contexts in which these conversations take place.

Analyzing large-scale social media data presents several challenges. Researchers must navigate high data volumes, the evolving nature of digital texts, user behaviors, data legitimacy, and ethical considerations when handling sensitive content [28,29]. Future research can build on these findings by exploring how emotional tone and language use in mental health organizations’ messaging influence health literacy, attitudes, beliefs, and behaviors.

### Conclusions

This study investigates how mental health organizations leverage social media to shape public discourse, focusing on the themes and linguistic strategies that define their messaging and information shared. The findings reveal that these organizations primarily emphasize community, positive sentiment, and workplace-related discussions on well-being. Health communication plays a crucial role in shaping public understanding and engagement, the clarity, accessibility, and trustworthiness of these messages are essential. Effective communication not only informs but also empowers individuals by providing actionable, evidence-based information. By analyzing these digital messages and the information shared, this study contributes to a deeper understanding of how mental health is framed on social media, offering insights into how messaging strategies can be refined to enhance public trust, reduce stigma, and promote meaningful engagement with mental health information.

## Supplementary material

10.2196/48230 Multimedia Appendix 1 List of accounts queried from X (formerly known as Twitter) API.

## Abbreviations

CERC: Crisis and Emergency Risk Communication
LIWC: Linguistic Inquiry and Word Count
RGB: red, green, and blue
RQ: research question
WHO: World Health Organization
