# Supplementary material for: Messaging and Information in Mental Health Communication on Social Media: Computational and Quantitative Analysis
Source: JMIR Infodemiology. 2025 Jul 3;5:e48230. doi: 10.2196/48230 (PMC12244273; doi:10.2196/48230)
Supplement: Multimedia Appendix 1 [file infodemiology-v5-e48230-s001.docx]

**Multimedia Appendix 1**

**List of Accounts Queried from Twitter API**

| **Account** | **Name** | **Tweets** |
| --- | --- | --- |
| @work_mental | Workplace Mental Health | 742 |
| @MHFAEngland | Mental Health First Aid England | 29513 |
| @NIMHgov | Mental Health NIMH | 9850 |
| @TimetoChange | Mental Health Awareness | 31856 |
| @mentalhealth | Mental Health Foundation | 46867 |
| @mindcharity | Mind | 78443 |
| @Rethink | Rethink Mental Illness | 19402 |
| @CharitySane | SANE | 44428 |
| @TogetherMW | TogetherMentalHealth | 5482 |
| @MHChat | Mental Health Chat | 94475 |
| @GPTW_UK | Great Place to Work(R)UK | 5787 |
| @GPTW_US | Great Place to Work US | 24975 |
| @healthyworknow | Healthy Work Campaign | 3564 |
| @HappyHealthyPod | The Happy Healthy Workplace | 318 |
| @healthlinksnews | Health Links | 2876 |
| @getwellright | Well Right | 792 |
| @CHWENews | Center for Health, Work & Environment | 3224 |
